# Factors associated with clinician willingness to adopt HPV self-sampling and self-testing for cervical cancer screening

**DOI:** 10.1017/cts.2024.604

**Published:** 2024-09-16

**Authors:** Luke Brennan, Tiwaladeoluwa Adekunle, Monica Kasting, Michele R. Forman, Victoria Champion, Natalia M. Rodriguez

**Affiliations:** 1 Weldon School of Biomedical Engineering, College of Engineering, Purdue University, West Lafayette, IN, USA; 2 Formerly at Brian Lamb School of Communication, Purdue University, West Lafayette, IN, USA; 3 Department of Public Health, College of Health and Human Sciences, Purdue University, West Lafayette, IN, USA; 4 Cancer Prevention and Control Program, Indiana University Simon Comprehensive Cancer Center, Indianapolis, IN, USA; 5 Formerly at Department of Nutrition Science, College of Health and Human Sciences, Purdue University, West Lafayette, IN, USA

**Keywords:** HPV testing, self-sampling, self-testing, cervical cancer, cancer screening

## Abstract

**Background:**

Cervical cancer screening rates in the USA fall behind national targets, requiring innovation to circumvent screening barriers. Cervical cancer screening where human papillomavirus (HPV) testing is performed on vaginal samples collected by the patients themselves (self-sampling) are effective and acceptable, and patient-operated rapid HPV tests (self-testing) are currently under development. It is unclear why there is ambivalence toward HPV self-sampling and self-testing among clinicians, an important stakeholder group. We conducted a mixed convergent quantitative and qualitative study to identify the factors influencing clinicians’ attitudes toward self-sampling and self-testing.

**Methods:**

A survey of Midwest clinicians distributed by professional group media and a market research firm between May and November 2021 was analyzed (*n* = 248) alongside in-depth interviews with Midwest clinicians from professional groups (*n* = 23). Logistic regression models examined willingness to support self-sampling and self-testing across respondent characteristics.

**Results:**

We report that family practice physicians and those in rural areas were more willing to adopt HPV self-sampling (adjusted OR (aOR) = 3.16 [1.43–6.99]; aOR = 2.17 [1.01–4.68]). Clinician willingness to support self-testing was positively associated with current use of self-testing for other conditions and negatively associated with performing 10 or more monthly cervical cancer screenings (aOR = 2.02 [1.03–3.95], aOR = 0.42 [0.23–0.78]). Qualitative data contextualize how clinical specialty and experience with self-sampling and self-testing for other conditions inform clinician perspectives.

**Conclusion:**

These data suggest clinician populations most accepting of initiatives to implement self-sampling and self-testing for cervical cancer screening and highlight that experience with other forms of self-testing could facilitate more widespread adoption for cervical cancer.

## Introduction

In the United States, only 73.9% of women with a cervix received cervical cancer screening from 2019 to 2021, falling below the Healthy People 2030 goal of 79.2% and leaving an extrapolated 19 million insufficiently screened [1]. Screening is an important and cost-effective secondary cervical cancer prevention strategy, as early detection of its biomarkers, human papillomavirus (HPV) or atypical cytology, can provide the opportunity to prevent or treat cervical cancers in low-grade, highly treatable, stages [2– 4]. In the USA, medically underserved subpopulations including immigrants and people without insurance have lower screening rates than the majority population [5,6]. Cervical cancer incidence has been increasing among non-Hispanic White women in low-income counties (1.0%/year, 95% CI 0.1%–4.5%) [2]. Likewise, racial/ethnic disparities are persistent in screening, incidence, and mortality rates of cervical cancer. Black women and Asian women were less likely to have received a Papanicolaou test (pap test) in the last 5 years (OR 0.40; 95% CI 0.22–0.89, *P* = 0.02) [3] or 3 years (OR 0.53; 95% CI 0.39–0.73)[4] respectively. Hispanic women have a disproportionately high cervical cancer incidence rate, and non-Hispanic Black women have a disproportionately high mortality rate [7].

The pap test, the traditional approach for cervical cancer screening, is associated with barriers including patients’ procedural anxiety, discomfort, and embarrassment, particularly if a male physician is performing the procedure [8,9]. More recently, high-risk HPV (hrHPV) testing where patients collect their own cervicovaginal samples using a kit and then send the preserved sample to a laboratory for analysis, called self-sampling, has emerged as an effective alternative to the more invasive and time-intensive pap smear [10]. Self-sampling mitigates some existing barriers to screening for vulnerable populations by presenting a convenient and private alternative to the pap smear as the primary screening method [11,12].

The increased adoption of self-sampling could allow for a next step in accessible testing where patients collect their own sample and also run that sample on a rapid HPV test themselves, called self-testing. This is possible because the nucleic acid amplification technology that allows laboratory tests to sensitively detect HPV even in most self-collected samples [10] is recently being implemented in at-home tests [13]. Only at-home tests that are invalid or positive for hrHPV would require a provider-collected sample be sent to a laboratory for cytology. Although HPV self-sampling and/or self-testing could provide promising ways of broadening access to cervical screening, understanding stakeholders’ perspectives is critical to effective implementation [14].

A systematic review and meta-analysis of studies across multiple nations demonstrated that women preferred this HPV self-sampling to clinician sampling [15]. In particular, a meta-review in the USA [5] and Europe [6] showed that women who do not respond to mail or telephone invitations for cervical screening were shown to be over twice as likely to respond to a mailed a self-sampling device rather than an invitation for a provider-collected sample. A study by Gupta *et al*., (2018) also found that over 90% of 130 patients preferred HPV self-sampling to clinician-led sampling [7]. Another study by Le *et al*., (2022) demonstrated that healthcare providers had some reservations about mail-based HPV self-sampling, such as concerns about follow-up, or patients failing to return the kits [17]. HPV self-testing was generally well accepted in a qualitative study of Appalachian women and providers, with providers hoping to screen patients who are overdue and patients hoping to ease the logistical barriers of screening [8]. In another study of American clinicians, 80% knew what “point of care” testing meant, and 97% were interested in adopting a point-of-care test for cancer screening in their practice. Providers were interested in the tests for different reasons and had differing preferences about how to communicate positive results to their patients [9]. Finally, a qualitative study with healthcare providers and women found that HPV self-sampling was generally acceptable to healthcare providers, but unlike the women participants they preferred for HPV self-test results to be received at the clinic [12].

Our recent study on Indiana providers’ attitudes toward screening innovations found that slightly less than half of the respondents (48%) were willing to support adopting patient self-testing due to its perceived limitations [16]. Specifically, these limitations included concerns about primary HPV testing limiting the ability for cellular changes to the cervix to be detected directly, as well as the perceived drawbacks of testing for a risk factor as opposed to the disease itself. This work builds upon the findings of this study and aims to identify which clinicians would try these testing methods.

Clinicians constitute a key group whose attitudes, beliefs, and practices are consequential for primary and secondary HPV prevention [18,19]. Understanding the factors relevant to clinicians’ receptivity toward inclusive and accessible screening methods such as HPV self-sampling and self-testing is essential to successfully implement these methods in clinics and benefit patients. This paper thus utilizes a convergent mixed-methods approach combining a survey and interviews to address the need for evidence on factors that influence clinicians’ acceptance of novel screening modalities. By doing so, it aims to uncover these factors and provide evidence supporting their rationale. This insight could then be used to develop campaigns aimed at promoting the adoption of these methods. The overarching research question for this study is as follows: which clinician characteristics are associated with willingness to adopt HPV self-sampling and self-testing?

## Methods

### Data collection

This study utilized a convergent mixed-methods approach that included a survey and in-depth interviews. Midwest clinicians who performed cervical cancer screening on at least one asymptomatic average-risk woman aged 21–65 years in the past month were eligible for the survey or interview. The IRB-approved, cross-sectional survey questionnaire was administered in two waves from May to November 2021. Analysis focused on the US Midwest, as a small enough area of interest to be characterized as one conceptual group, while still large enough to obtain statistical power. The first wave recruited 76 participants across Indiana, Kentucky, Ohio, and Michigan through emails to local healthcare organizations, professional groups, and the American College of Obstetricians and Gynecologists (ACOG) district V newsletter. The second wave recruited another 195 Indiana clinicians through the market research firm Dynata to reach providers of diverse specialties and backgrounds. (Purdue IRB-2019–132; IRB-2021–12; IRB-2021–617). This survey items included respondent demographics and clinical practice information, screening modalities, and attitudes toward HPV self-testing and HPV self-sampling. Survey items regarding new screening methods were posed in the context of “a 35-year-old asymptomatic patient that had a normal last screening test (normal Pap/HPV-negative) 5 years ago, like all of her previous screening tests.” This conservative case was used as it reflects standard preventive screening which applies to most patients and excludes scenarios which would put additional demands on the method proposed.

At-home *self-sampling* was introduced with the following description:

Self-sampling for cervical cancer screening allows women to collect their own vaginal swab in private with an FDA-approved self-collection device and instructions. The self-collected samples can be collected in the home, workplace, or elsewhere and then sent to the laboratory by dropping it off in their mailbox, at the clinic, or given to a community health worker.

Respondents were asked to rate their agreement with the following question from strongly agree to strongly disagree: “I would support offering HPV self-sampling to my patients, where a woman collects her own specimen at home without needing to come to the clinic if the results are normal.”

At-home *self-testing* was introduced with the following description:

An HPV rapid diagnostic test like those being developed for point-of-care testing could also enable at-home testing if they were simple enough for patients to use (other examples of at-home testing include pregnancy tests and blood glucose tests). This would be called an at-home rapid HPV test. As opposed to self-sampling at home, at-home rapid HPV tests would also deliver results at home within a matter of minutes.

Respondents were asked to rate their agreement with the following question from strongly agree to strongly disagree: “I would support offering at-home rapid HPV testing for my patients to complete without needing to come into the clinic if the results are normal.”

All respondents took the same survey via Qualtrics and were compensated upon completion with a $25 reimbursement as an electronic gift card or Dynata reward points.

A 30–60-minute interview protocol was created using the diffusion of innovation theoretical framework, covering the same topics as the survey questionnaire to contextualize and provide reasoning behind the findings from the quantitative survey. The questionnaire was reviewed for clarity and scope by public health professors at Purdue with expertise in HPV care delivery, as well as clinicians in the OB/GYN field at Indiana University. Interview participants were recruited for follow-up zoom interviews through emails sent to the researchers’ networks of Indiana clinicians, in addition to survey respondents who consented to being contacted for a follow-up interview. Interviews continued until new themes and new perspectives stopped emerging in successive interviews, which occurred after 23 clinician interviews.

### Data analysis

#### Quantitative analysis

The outcome variables of this study were (1) respondents’ willingness to support adoption of at-home HPV self-sampling for their clinic and (2) respondents’ willingness to support at-home HPV self-testing as described above, each of which was dichotomized as “Strongly agree” or “Agree” versus “Uncertain,” “Disagree,” or “Strongly disagree.” Logistic regression analyses were performed separately for each outcome variable to examine the association with clinician and clinic characteristics. Similar respondent groups were combined (e.g., obstetrician/gynecologist and gynecologic oncologist) or eliminated (e.g., 4 clinical training “other” respondents) to facilitate meaningful comparisons between groups relevant to our research questions, with high power and reasonable sample sizes. Collinearity among variables was assessed before regressions, finding no variables to have a generalized variance inflation factor (gVIF) above 2. Bivariable logistic regression was used to generate odds ratios (ORs) with 95% confidence intervals (CIs), multivariable logistic regression generated Wald statistic-based adjusted ORs (aORs), CIs, and p-values for each regressor. Finally, variables that were included in a reduced model using forward stepwise multivariable logistic regression are also reported. Data analyses were performed in SPSS v26.

#### Qualitative analysis

We explored qualitative data to contextualize insights related to the quantitative findings of this study, and some of the predictors identified in the quantitative analysis emerged as salient themes in our qualitative research. We developed a codebook by analyzing interviews for broad themes related to the interview guide. Following the initial thematic analysis [16], qualitative analysis was integrated with quantitative findings by examining the themes that correspond to regression factors which demonstrated strong correlations with adoption of self-sampling or self-testing. Any consensus opinions, barriers, facilitators, and dissonance within each theme were reported.

## Results

### Quantitative sample

A total of 271 clinicians were recruited from the Midwest USA to take the survey. For descriptive characteristics, groups that represented 5% or less of the total sample (this included 5 “prefer not to answer” and 1 “Transgender Female” respondents from the sex variable, 4 “other” respondents to the clinical training variable, and 13 “other” respondents in the clinical specialty variable) were excluded from the final analyses to increase statistical power, leaving *n* = 248 respondents included in quantitative analyses. The survey consisted of 77 items and took an average of 15 minutes to complete. As Table 1 shows, the majority of the respondents are non-Hispanic White (*n* = 190, 76%), followed by Hispanic/Latinx (*n* = 20, 8%), non-Hispanic Black (*n* = 13, 5%), Asian (*n* = 14, 5%), and individuals who identified as “other” (*n* = 11, 4%). Providers identified specialties as family medicine (*n* = 162, 65%), obstetrics/gynecology (*n* = 51, 20%), and internal medicine (*n* = 35, 14%). The largest proportion of providers primarily served patients using Medicaid (*n* = 109, 43%), while a smaller proportion primarily served privately insured/HMO patients (*n* = 91, 36%). The smallest proportion of clinicians served patients with self-pay and other forms of insurance or were unsure about their patients’ insurance (*n* = 48, 19%).

**Table 1.** Survey respondent demographics (*N* = 248)

Item	Value (percent) mean, stdev
*Sex:	
Female	175 (70%)
Male	73 (29%)
**Clinical specialty:	
Family medicine	162 (65%)
Gynecologic specialties	51 (20%)
Internal medicine	35 (14%)
**Federally Qualified Health Center (FQHC) status:**	
No/Unsure	167 (67%)
Yes	81 (32%)
**Majority patient payment method:**	
Medicaid	109 (43%)
Private insurance/HMO	91 (36%)
Uninsured/Self–pay/Unsure/Other	48 (19%)
**Monthly cervical cancer screenings performed:**	
Ten patients or more	155 (62%)
Less than 10 patients	93 (37%)
**Familiarity with patient self-sampling for any purpose:**	
Do not currently use	175 (70%)
Currently use	73 (29%)
**Familiarity with at-home self-testing for any purpose:**	
Do not currently use	171 (68%)
Currently use	77 (31%)
***Clinical training:	
Physician	134 (54%)
Advanced practice professionals	114 (45%)
**Race/ethnicity:**	
White, non–Hispanic	190 (76%)
Hispanic/Latinx	20 (8%)
Asian	14 (5%)
Black, non–Hispanic	13 (5%)
Other	11 (4%)
**Clinic type:**	
Group practice	99 (39%)
Community health clinic	71 (28%)
Hospital	38 (15%)
Private practice	30 (12%)
Other	10 (4%)
**Clinic setting:**	
Suburban	113 (45%)
Urban	87 (35%)
Rural	48 (19%)
**Years in practice (including residency):**	14.2, 10.4
**Primary HPV testing is an effective cervical cancer screening method:**	
True	139 (56%)
False/Uncertain*	109 (43%)
**Support adopting a point-of-care HPV test in clinic:**	
Strongly agree, Agree	206 (83%)
Uncertain, Disagree, Strongly Disagree	42 (16%)
**Support offering HPV self-sampling:**	
Strongly agree, Agree	129 (52%)
Uncertain, Disagree, Strongly Disagree	119 (47%)
**Support offering at-home rapid HPV testing:**	
Strongly agree, Agree	126 (50%)
Uncertain, Disagree, Strongly Disagree	122 (49%)

HPV = human papillomavirus.

* Gender: excluded six “Prefer not to answer” and one “Transgender Female” respondents.

** Clinical training: excluded 4 “Other” respondents, and “advanced practice professionals” includes 5 physician assistants and 109 nurse practicioners

*** Clinical specialty: excluded 13 “Other” specialties.

### Qualitative sample

A total of 23 clinicians completed a qualitative interview, which lasted 30–60 minutes. Our sample of interview participants reflected similar demographics to the quantitative survey participants, with the majority of clinicians being White (*n* = 15, 65%), Female (*n* = 14, 60%), and family medicine specialists (*n* = 12, 52%) (Table 2).

**Table 2.** Interview participant demographics (*N* = 23)

Demographic	Total
	**23**
**Gender:**	
Female	14 (61%)
Male	4 (17%)
Unknown/Prefer not to answer	5 (22%)
**Race/ethnicity:**	
White, non-Hispanic	15 (65%)
Other	8 (35%)
**Clinical training:**	
Physician	14 (61%)
Nurse practitioner	9 (39%)
**Clinical specialty:**	
Family medicine	12 (52%)
Other*	4 (17%)
Obstetrician/gynecologist	4 (17%)
Internal medicine	2 (8%)
Gynecologic oncologist	1 (4%)
**Federally Qualified Health Center (FQHC) status:**	
No	14 (60%)
Yes	7 (30%)
Unsure	2 (10%)

### Results for self-sampling

More than half (52%, *n* = 129) of the clinician sample expressed support for self-sampling at home. Willingness to support the adoption of at-home self-sampling was independently, positively associated with clinical specialty in family medicine (aOR 3.16 [95% CI 1.43–6.99]) versus gynecologic specialties, and rural clinic setting versus suburban (aOR 2.17 [95% CI 1.01–4.68]). Of all the variables in the multivariable model (Table 3), the stepwise regression model included the variables of clinical specialty (aOR 2.76 [95% CI 1.35–5.65] for family medicine and aOR 1.52 [95% CI 0.58–3.99] for internal medicine vs gynecology), less than 10 monthly cervical cancer screenings performed (aOR 0.61 [95% CI 0.34–1.09]), familiarity with patient self-sampling for any purpose (aOR 1.64 [95% CI 0.85–3.15]), familiarity with at-home testing for any purpose (aOR 1.84 [95% CI 0.97–3.47]), and clinic setting (aOR 1.95 [95% CI 0.94–4.05] for rural or aOR 1.54 [95% CI 0.84–2.45] for urban vs suburban).

**Table 3.** Provider characteristics and willingness to support at-home self-sampling

	Bivariable model	Full multivariable model	Reduced multivariable model
	OR (95% CI)	aOR (95% CI)	aOR (95% CI)
**Gender:**			
Female	ref	ref	
Male	1.27 (0.73–2.19)	1.41 (0.66–2.98)	
**Clinical specialty:**			
Gynecologic specialties	ref	ref	ref
Family medicine	**2.45 (1.28–4.69)**	**3.16 (1.43–6.99)**	**2.76 (1.35–5.65)**
Internal medicine	1.12 (0.46–2.71)	1.42 (0.50–4.03)	1.52 (0.58–3.99)
**Federally Qualified Health Center (FQHC) status:**			
No/Unsure	ref	ref	
Yes	1.24 (0.72–2.10)	1.13 (0.53–2.42)	
**Majority payment method:**			
Private insurance/HMO	ref	ref	
Medicaid	0.87 (0.50–1.51)	0.68 (0.32–1.43)	
Uninsured/Self–pay/Unsure/No definable payment majority/Other*	0.75 (0.37–1.52)	0.85 (0.37–1.96)	
**Monthly cervical cancer screenings:**			
10 patients or more	ref	ref	ref
Less than 10 patients	0.79 (0.47–1.33)	0.65 (0.36–1.20)	0.61 (0.34–1.09)
**Familiarity with patient self-sampling for any purpose:**			
Don’t currently use	ref	ref	ref
Currently use	**2.23 (1.26–3.93)**	1.89 (0.93–3.83)	1.64 (0.85–3.15)
**Familiarity with at-home testing for any purpose:**			
Don’t currently use	ref	ref	ref
Currently use	**2.34 (1.34–4.09)**	1.79 (0.93–3.46)	1.84 (0.97–3.47)
**Clinical training:**			
Physicians	ref	ref	
Advanced practice professionals*	0.86 (0.52–1.42)	0.78 (0.37–1.66)	
**Race/ethnicity:**			
White, non-Hispanic	ref	ref	
Asian	0.66 (0.22–1.98)	0.58 (0.17–2.05)	
Black, non-Hispanic	1.03 (0.33–3.17)	1.44 (0.39–5.29)	
Hispanic/Latinx	0.88 (0.35–2.21)	0.65 (0.22–1.90)	
Other*	0.73 (0.22–2.49)	1.03 (0.26–4.05)	
**Practice type:**			
Group practice*	ref	ref	
Community health clinic	1.08 (0.59–2.00)	0.91 (0.39–2.16)	
Hospital*	0.94 (0.45–1.99)	1.17 (0.46–2.98)	
Other	0.94 (0.26–3.46)	0.80 (0.19–3.45)	
Private practice	1.08 (0.47–2.44)	0.89 (0.36–2.21)	
**Clinic setting:**			
Suburban	ref	ref	ref
Rural	1.89 (0.95–3.77)	**2.17 (1.01–4.68)**	1.95 (0.94–4.05)
Urban	1.27 (0.73–2.22)	1.54 (0.79–3.01)	1.54 (0.84–2.83)
**Years practicing (including residency):**	0.99 (0.97–1.01)	0.98 (0.95–1.01)	
**Primary HPV testing is an effective cervical cancer screening method:**			
False/Uncertain	ref	ref	
True	1.19 (0.72–1.97)	1.37 (0.77–2.45)	

* Collapsed answer choices.

Gender: excluded five “prefer not to answer” and one “Transgender Female” respondents.

Clinical training: excluded four “other” respondents.

Clinical specialty: excluded 13 “other” respondents.

HPV = human papillomavirus.

### Results for self-testing

Half (50%; *n* = 126) of the clinician sample expressed support for self-testing at home (Table 4). Willingness to support the adoption of at-home self-testing was independently, positively associated with currently using at-home testing for any purpose (aOR 2.02 [95% CI 1.03–3.95]) and negatively associated with fewer than 10 monthly cervical cancer screenings (aOR 0.42 [95% CI 0.23–0.78]). The reduced model included respondent sex (aOR 1.82 [95% CI 1.03–3.21] for males vs females), performing fewer than 10 cervical cancer screenings per month (aOR 0.64 [95% CI 0.38–1.09]), and current use of at-home testing for any purpose (aOR 1.90 [95% CI 1.09–3.32]).

**Table 4.** Provider characteristics and willingness to support at-home self-testing

	Bivariable model	Full multivariable model	Reduced multivariable model
	OR (95% CI)	aOR (95% CI)	aOR (95% CI)
**Gender:**			
Female	ref	ref	ref
Male	1.72 (0.99–3.00)	2.01 (0.95–4.24)	**1.821 (1.03–3.21)**
**Clinical specialty:**			
Gynecologic specialties	ref	ref	
Family medicine	1.35 (0.72–2.55)	1.89 (0.87–4.13)	
Internal medicine	2.23 (0.92–5.39)	2.78 (0.97–7.97)	
**Federally Qualified Health Center (FQHC) status:**			
No/Unsure	ref	ref	
Yes	0.79 (0.47–1.35)	0.71 (0.33–1.52)	
**Majority payment method:**			
Private insurance/HMO	ref	ref	
Medicaid	1.00 (0.58–1.75)	1.48 (0.70–3.13)	
Uninsured/Self–pay/Unsure/No definable payment majority/Other*	1.31 (0.65–2.65)	1.61 (0.69–3.74)	
**Monthly cervical cancer screenings:**			
10 patients or more	ref	ref	ref
Less than 10 patients	0.65 (0.39–1.09)	**0.42 (0.23–0.78)**	0.641 (0.38–1.09)
**Familiarity with patient self-sampling for any purpose:**			
Don’t currently use	ref	ref	
Currently use	1.16 (0.67–2.01)	1.20 (0.59–2.44)	
**Familiarity with at-home testing for any purpose:**			
Don’t currently use	ref	ref	ref
Currently use	**1.82 (1.05–3.16)**	**2.02 (1.03–3.95)**	**1.90 (1.09–3.32)**
**Clinical training:**			
Physicians	ref	ref	
Advanced practice professionals*	0.88 (0.54–1.46)	1.66 (0.79–3.48)	
**Race/ethnicity:**			
White, non–Hispanic	ref	ref	
Asian	0.73 (0.25–2.20)	0.54 (0.15–1.92)	
Black, non-Hispanic	1.14 (0.37–3.53)	1.64 (0.44–6.10)	
Hispanic/Latinx	1.20 (0.47–3.02)	1.49 (0.50–4.45)	
Other*	1.17 (0.35–3.98)	1.19 (0.30–4.81)	
**Practice type:**			
Group practice*	ref	ref	
Community health clinic	0.59 (0.32–1.08)	0.45 (0.19–1.06)	
Hospital*	1.23 (0.57–2.63)	1.16 (0.46–2.94)	
Other	0.34 (0.08–1.40)	0.31 (0.07–1.46)	
Private practice	0.80 (0.35–1.81)	0.69 (0.28–1.71)	
**Clinic setting:**			
Suburban	ref	ref	
Rural	1.28 (0.65–2.51)	1.43 (0.68–3.02)	
Urban	1.51 (0.86–2.65)	1.76 (0.91–3.42)	
**Years practicing (including residency):**	1.00 (0.98–1.03)	1.01 (0.98–1.04)	
**Primary HPV testing is an effective cervical cancer screening method:**			
False/Uncertain	ref	ref	
True	0.90 (0.54–1.49)	0.83 (0.46–1.47)	

* Collapsed answer choices.

Gender: excluded five “prefer not to answer” and one “Transgender Female” respondents.

Clinical training: excluded four “other” respondents.

Clinical specialty: excluded 13 “Other” respondents.

### Qualitative insights on factors associated with willingness to adopt self-sampling and self-testing

Two of the significant factors from the quantitative analysis, provider specialty and familiarity with self-sampling and self-testing, also emerged as salient in the qualitative analysis. Another factor, gender, was significant for support of self-testing but not self-sampling and was found to play an important role in screening through our qualitative analysis. Below, qualitative insights on clinician specialty and familiarity with self-sampling/self-testing as well as gender are described in further detail.

### Clinician specialty

In quantitative analyses, clinicians who specialized in family medicine were 3.16 times more likely to adopt self-sampling. Qualitative insights on how current screening methods play a part of clinicians’ broader workflow provided further context for this result. On the one hand, patients who come in for other reasons may elect not to get a pap smear. As one family medicine practitioner described: “*if they’re coming in, like she came in for diabetes… that wouldn’t be like “oh by the way you’re due for a pap smear. Can we do one today?…”and when they say…’no,’ maybe six times out of ten*.” On the other hand, patients that call the clinic for pap smears may then not receive other types of care: “*A lot of times for women, their first appointment they call and they say “oh I, you know, I need my pap smear…. our practitioner, you know, does a pap smear and maybe a mammogram is appropriate, and like that counts as their new patient visit,, but then they’re like, ‘oh, for all your other medical problems follow up with your family physician.’ Then after that they do not really have like dedicated time kind of built in…*”

### Familiarity with self-sampling and self-testing

Qualitative interviews supported the finding that providers who have previously used self-sampling or self-testing for another purpose were more likely to be willing to adopt each method for cervical cancer screening, as clinicians’ experience with other self-sampling methods such as the Fecal Immunochemical Test for colorectal cancer, shaped their perceptions of self-sampling for HPV.

Clinicians who had positive experiences with other methods of self-testing were more inclined to support HPV self-sampling. As one clinician shared: “*I mail them…the screening test for colon cancer… they send it they go back to the lab and results come in. So it would be no different to me*.” The same provider reported that he had no concerns about self-collected samples due to his experience with this test: “*…nobody watches you collect poop for the FIT test and I believe the test results, right? So, I think it would be sort of, you know, similar*”

However, some clinicians had previously experienced challenges with follow-ups from self-sampling. These challenges with prior experiences informed their perspectives of self-sampling for HPV. As one provider described: “*I don’t know if I’m too keen on the self-sampling. We do self-sampling for like FOBT screening for …colon cancer and things like that. And a lot of times …. They don’t collect the sample appropriately. They never bring the sample back in….And so, I don’t know if I’m okay with the home sampling, the rapid getting the results back, eh if it’s just HPV, but I still feel like it leads us [to] still not doing that complete physical exam and complete well-woman’s exam, where were able to make sure, okay, it’s just HPV, but there’s no other high-grade cells that was found…*”

### Gender

Qualitative interviews provided some insights into the importance of gender in clinician’s willingness to adopt innovations in cervical cancer screening. Male participants elaborated on the discomfort some of their patients experience with receiving pap tests from them. This anticipated/actual patient discomfort with gender discordance during pap tests might contribute to male providers’ willingness to support self-sampling and or self-testing. It should be noted that only self-testing and not self-sampling showed statistically significant support in the quantitative data. While male providers expressed familiarity and comfort with pap tests, some reported that their gender contributed to patients’ discomfort with getting pap smears. As one male provider shared: “*I think young women are told from toddler and forward, do not let strange old man look at your bottom*.” He also shared that this discomfort occasionally leads patients, particularly those under 21 to refuse HPV screening: “*Occasionally a woman will say, I will not be seen by a male provider. Okay, okay, and we say, I cannot change my gender.”* This provider shared that one of the benefits he anticipated for self-testing would be increased testing: *“I would hope that the benefit would be that many more people would be tested*.”

Cultural beliefs and attitudes are also relevant to the role of gender in screening. Another male provider shared how his Somali patients were historically reluctant to be seen by a male provider: “*there were some cultural things, but, you know, especially Somali not wanting to be seen by a male…when I first started would not want to be seen in the same building with a male*.”

Interviews with female providers also offered additional insight into gender-related factors/barriers in pap smears conducted by male providers: “*in a couple of practices that I was looking at…the majority of preceptors were men and older men who just didn’t do [cervical cancer screening] very often and so it just wasn’t a priority. So I think, you know, that’s one of the issues is that if you are a woman patient coming to a physician, and you’re seeing somebody who isn’t comfortable doing paps and pelvics in their office, then they have to send you on to another person and that’s another fee and that’s another time and time off of work and it slows down the process…*.” Another female provider shared that male residents at her hospital/clinic prefer not to do pap tests: “*Sometimes our residents opt for the like easier thing for them to do which would be to just let the patient self-swab, and you might lose some of our like follow up with patients… I find with some of our male providers they prefer not to do pap tests and so they might just let the patient self-swab and sort of end the screening and workup at that point”*

Thus, gender is relevant to the traditional screening process for cervical cancer, with both male and female clinicians describing some patients’ discomfort with getting screened by male clinicians, as well as perceived discomfort from [some] of their male colleagues with performing pap tests. This perceived discomfort in gender discordant patient/provider pairings may inform the quantitative findings on male providers’ willingness to support self-testing.

## Discussion

Clinicians are a key stakeholder group for the implementation of novel approaches to screening [17]. This study addressed the gap in literature about factors that influence clinicians’ willingness to adopt HPV self-sampling and self-testing, using data from a survey and in-depth interviews in a mixed-methodological approach.

Analysis of quantitative data found that respondents who screen frequently were generally more willing to support adoption of self-sampling and self-testing for cervical screening, indicating potential utility as a high-throughput way of performing many daily screenings. Alternatively, this indicates that self-testing may not serve to increase screenings from clinicians who screen infrequently, which was a potential value proposition of such methods. Clinicians who currently use self-testing for other diseases were supportive of self-testing for cervical cancer. This indicates that previous experiences with self-testing do not seem to dissuade many clinicians from using it for cervical cancer. This likely reflects good experiences with self-testing. Finally, while male providers were more likely to be willing to adopt self-testing, this was not the case for self-sampling. This finding is in accord with other studies in which Midwest male providers were not significantly interested in HPV self-sampling for patients who are regularly screened (though they were interested for patients who were overdue for screening) [25]. This finding underscores distinct considerations for the adoption of self-sampling as compared to self-testing, and future research may further explore why clinicians’ willingness to adopt self-testing may differ from self-sampling.

HPV self-sampling has been found to be an effective screening modality for cervical cancer [10]. Although HPV self-sampling has been found to be acceptable to women [15] and is preferred over clinician sampling for privacy and ease of use [11], previous studies have found more ambivalence from healthcare providers who see HPV self-sampling as beneficial for patients but have concerns about the logistical challenges related to returning the test and lost follow-up [17]. This study found that practicing family medicine and practicing in a rural setting were each independently, positively associated with supporting adoption of self-sampling in the full multivariable logistic model. Likewise, performing more than 10 monthly cervical cancer screenings and current use of at-home testing were positively associated with supporting adoption of at-home self-testing.

Qualitative insights on specialty-related differences in willingness to adopt HPV self-sampling suggest that these differences may be related to the varying responsibilities and work streams of practitioners depending on specialty. Specifically, family medicine practitioners shared that patients that come in for screenings may not receive other types of care, and patients that come in for other types of care may not get screened. This finding supports existing literature on specialty-related differences in HPV screening practices [20–22]. An earlier study on HPV screening practices by specialty found that obstetrician/gynecologists were the most likely to perform HPV screenings in asymptomatic patients [20]. Furthermore, this study found that most physicians who did not screen for sexually transmitted disease including HPV were in pediatrics, internal medicine, and family medicine. Likewise, another study found specialty-related differences in attitudes toward co-testing, with women’s health nurse practitioners being more likely than family nurse practitioners to believe that co-testing provides assurance for patients and being more likely to routinely use co-testing [21]. Specific to HPV self-sampling, a Canadian study found that primary care providers were more likely than obstetrician/gynecologists to agree that HPV self-sampling helped ease the process of screening for patients. This study also found an increased (but statistically insignificant) likelihood of obstetrician/gynecologists indicating concerns about follow-up [22]. Our study thus further demonstrates that the specialties of clinicians play an important role in their attitudes toward various modes of cervical cancer screening, including HPV self-sampling.

Furthermore, our report that clinicians who practice in rural areas were more likely to be willing to adopt HPV self-sampling may be explained by the documented disparities in cervical cancer screening for individuals in rural areas and the barriers that exist at the provider, facility, and systems levels [23,24]. A study in rural Appalachia found that 33% of participants had not received cervical cancer screening for 5 years or more [23]. A lack of facilities is one of the barriers that contributes to disparities in screening access. Clinicians in these areas may understand the potential of HPV self-sampling in mitigating barriers, particularly those related to geographical distance, and increasing screening access.

In this study, current use of self-testing for other diseases is related to clinicians’ willingness to adopt HPV self-testing. Additionally, interviews with clinicians demonstrated that their negative and positive experiences with other self-sampling mechanisms in the past shaped their attitudes toward self-sampling and self-testing in the context of HPV. Clinicians with previous positive experiences with other methods of self-testing such as FIT or COVID were receptive to HPV self-sampling, and clinicians with previous negative experiences had more reservations about the application of self-sampling to cervical cancer screening. Familiarity with self-testing in other contexts is thus relevant to willingness to adopt self-testing for HPV. This study thus stresses that familiarity with self-sampling as another important dimension for consideration in clinicians’ willingness to adopt self-sampling for cervical cancer.

Additionally, this study highlighted the number of monthly cervical cancer screenings offered as a factor that may influence clinicians’ willingness to adopt HPV self-testing. Clinicians who perform more frequent screenings may be more familiar with the barriers to screening experienced in their patient population. More research is needed to understand how a provider’s frequency of cervical cancer screenings conducted influences their attitude toward HPV self-testing.

This study’s qualitative insights on the role of gender provide support for previous findings on the relevance of clinician gender for screening. Another study found that gender discordance between patients and healthcare providers was associated with lower rates of screening for cervical, breast, and colorectal cancer, even after adjusting for racial/ethnic concordance [26]. A study in Scotland found that male providers were more likely than female providers to consider general practitioners responsible for recommending and conducting prostate cancer screening, a disease that largely impacts males [27]. Likewise, another international study found that female providers were more likely to provide more preventive care and recommend cancer screening than male providers [28]. Our study demonstrated that these gender differences exist in clinicians’ willingness to adopt HPV self-sampling and self-testing. Qualitative insights further highlighted gender discordance as a barrier to screening, as male providers elaborated on patients’ discomfort with male provider screening, and female providers reported on some male clinicians’ discomfort with conducting screening. More research is needed to understand how gender shapes clinicians’ attitudes toward reproductive health screenings, particularly in the context of HPV self-testing and self-sampling.

In summary, this study underscores the importance of targeted strategies responsive to: (i) clinician specialty, (ii) rural/urban setting, (iii) volume of screenings performed, (iv) prior experience with self-sampling/self-testing, and (v) gender. Effective implementation of HPV self-testing and self-sampling technologies necessitates careful engagement with clinicians to address their concerns and demonstrate the utility of testing innovations to the expansion of screening access for underserved populations.

One limitation of this study is its reliance on clinicians’ self-report about their willingness to adopt these screening modalities. Clinicians’ self-reported willingness to adopt may differ substantially from their actual attitudes and behaviors in a real-world scenario. Furthermore, this study sampled clinicians from the US Midwest, which naturally limits its generalizability. Finally, although clinicians can be influential in decision-making around adoption of new technologies and screening modalities, system-wide policies or other institutional stakeholders such as patients, healthcare organizations’ leadership, and insurance companies will also play substantial roles.

## Conclusion

Widespread screening is a critical secondary prevention strategy towards the elimination of cervical cancer. Self-sampling is increasingly recognized as an effective means of mitigating barriers to screening, particularly for underserved populations who are less able or willing to attend an in-person clinic visit. While there is extensive support for the acceptability of self-sampling among patients, extant literature demonstrates that clinicians are ambivalent toward self-sampling and self-testing. This work contributes to the literature by highlighting the provider characteristics associated with willingness to adopt HPV screening innovations. By underscoring the association between prior experience with self-sampling and self-testing, gender, and specialty, this study reveals the need for targeted strategies for engaging providers and promoting HPV self-sampling and self-testing. Specifically, this study demonstrates that obstetrician/gynecologists, clinicians without prior experience with self-sampling/self-testing, and clinicians who perform screenings less frequently may need more compelling evidence that addresses their concerns to support the adoption of these technologies. Future studies might use these characteristics to explore feasible ways to address clinicians’ concerns about self-testing and self-sampling.

## Data Availability

To protect the privacy of participants, the qualitative data cannot be made available. Quantitative data is available upon request – please email corresponding author.
